# Repetition increases belief in climate-skeptical claims, even for climate science endorsers

**DOI:** 10.1371/journal.pone.0307294

**Published:** 2024-08-07

**Authors:** Yangxueqing Jiang, Norbert Schwarz, Katherine J. Reynolds, Eryn J. Newman

**Affiliations:** 1 School of Medicine and Psychology, The Australian National University, Canberra, ACT, Australia; 2 Mind and Society Center, University of Southern California, Los Angeles, California, United States of America; 3 Department of Psychology, University of Southern California, Los Angeles, California, United States of America; 4 Marshall School of Business, University of Southern California, Los Angeles, California, United States of America; 5 Melbourne Graduate School of Education, The University of Melbourne, Parkville, VIC, Australia; Yeditepe University, TÜRKIYE

## Abstract

Does repeated exposure to climate-skeptic claims influence their acceptance as true, even among climate science endorsers? Research with general knowledge claims shows that repeated exposure to a claim increases its perceived truth when it is encountered again. However, motivated cognition research suggests that people primarily endorse what they already believe. Across two experiments, climate science endorsers were more likely to believe claims that were consistent with their prior beliefs, but repeated exposure increased perceptions of truth for climate-science and climate-skeptic claims to a similar extent. Even counter-attitudinal claims benefit from previous exposure, highlighting the insidious effect of repetition.

## Introduction

Misinformation travels via many means. False claims may originate on fringe websites, be widely shared on social media, and repeated by mainstream media, often with the aim to provide “balanced reporting” that gives a voice to all sides. Unfortunately, the mere repetition of a claim can increase the degree to which people accept it as true [1, 2]. While this “illusory truth effect” (ITE) is well established, most of the evidence pertains to everyday knowledge and trivia. It remains unclear whether repetition also increases people’s assessments of truth when the content of repeated claims clashes with their own strongly held beliefs and attitudes. We address this question in a context that has received particular attention in discussions of “balanced reporting”, namely the repetition of claims that are incompatible with well-established insights of climate science [3–5]. Specifically, we test, (i) whether repetition increases the perceived truth of climate claims that are aligned with climate scientists vs. climate skeptics, (ii) whether the impact of claim repetition is moderated by recipients’ prior climate change beliefs, with a particular interest in (iii) whether repeating climate skeptic-aligned claims can increase their perceived truth even among recipients who are highly concerned about climate change. Whereas metacognitive theorizing suggests that repetition is likely to increase the perceived truth of climate-science and climate-skeptic aligned claims for all recipients, theorizing about motivated cognition suggests that recipients’ pre-exposure attitudes should limit the impact of counter-attitudinal claims. We address both lines of theorizing in turn.

### Metacognitive theorizing: The illusory truth effect

Metacognitive research shows that people attend to the content of a claim and the subjective experiences that accompany its processing when they evaluate its merits [6]. When information feels easy to process—a claim is easy to perceive, easy to imagine, or easy to retrieve—people judge it as more likely to be true [7]. Feelings of ease or difficulty can arise from attributes that provide valid information about a claim as well as from purely incidental influences. For example, a coherent and logically valid argument is easier to comprehend than an incoherent one and may warrant a higher rating of truth [8]. But an experience of easy processing can also arise from tangential influences that are not diagnostic of truth, such as the simple repetition of a claim. Repeated claims are processed more quickly, a classic (and robust) finding in repetition priming [9]. Because people are more sensitive to their feelings than to the source that elicited them, they usually draw on their momentary feelings when forming a judgment [10]. Hence, they use ease of processing as an informative cue to truth, regardless of whether the argument was coherent [11], has been repeated [12], or was simply presented in an easier to read color contrast [13–15]—whenever a claim is easy to process, it is more likely to “feel right” [6].

The power of mere repetition is captured in a classic paradigm from cognitive psychology named the illusory truth effect (ITE) [2, 12, 16]. In the ITE paradigm, people view a series of claims during an initial exposure phase. After a short delay, they rate the perceived truth of another series of claims, half of which they have seen during the exposure phase (repeated claims) and half of which are new (non-repeated). Which claims are repeated or new is counterbalanced across participants, allowing researchers to assess the impact of repetition on judged truth. Since its discovery in 1977 [12], the key finding is among the most robust results in cognitive psychology: a given claim is more likely to be judged true when it has been encountered before. Moreover, a single repetition is sufficient to produce this illusory truth effect [2].

Recent reviews have highlighted the robustness of the ITE across different contexts and domains of judgment. The tendency to believe repeated claims holds not only for unfamiliar claims, but also for claims that people can later identify as false on the basis of their general knowledge [17]. Informing people about the influence of repetition [18], warning them that some of the statements are false [19] or come from a low credibility source [20, 21], attenuates the ITE but does not eliminate it. ITE effects have been observed in many domains, including trivia statements [22], opinions of others [23], product-related claims [24, 25], strong and weak arguments [26], and fake news headlines [27]. However, little is known about the extent to which repetition affects people’s assessments of truth when claims are clearly counter-attitudinal and at odds with strongly held prior beliefs.

### Motivated cognition

In contrast, strongly held prior beliefs have received extensive attention in motivated cognition research. In this domain, researchers assume that people are likely to reject claims that run counter to their own beliefs and exhibit a disconfirmation bias [28–31]. Arguments that are inconsistent with recipients’ beliefs or political ideology are processed more slowly, as indicated by longer reading times, and elicit more counter-arguing [32–34], which limits their acceptance [35, for a review]. People also perceive counter-attitudinal claims as less accurate than pro-attitudinal claims that are aligned with their own position on a given topic [36, 37]. The observed resistance to counter-attitudinal arguments increases with attitude strength—that is, the extremity of one’s own position and the conviction with which it is held [38, 39].

If people’s truth assessment—in the present studies, their assessment of the perceived truth of climate-related claims—is largely guided by motivated reasoning, repeated exposure to counter-attitudinal claims that align with climate skeptics and climate deniers should not increase truth perceptions among people who endorse climate science.

### The present research

In light of these different perspectives, what might we expect when people who endorse climate science are repeatedly exposed to climate-skeptic claims? A motivational account suggests that mere repetition will do very little to shape their perceptions of truth. People will presumably attend to the content of the claims and reject them as inaccurate when they conflict with their own beliefs and attitudes. In contrast, a metacognitive account suggests that people also attend to how easily a claim can be processed and use this metacognitive experience as information in judging its likely truth. If so, climate science endorsers may (i) evaluate claims that are consistent with their beliefs as truer than claims that are not but may (ii) nevertheless evaluate repeated counter-attitudinal claims as truer than non-repeated ones.

We tested the influence of repetition on the perceived truth of pro- and counter-attitudinal claims in two experiments. In both studies, we showed people who endorsed climate science a series of climate related claims that were either aligned with climate science (pro-attitudinal) or with climate skepticism (counter-attitudinal). After a 15-minute delay, participants evaluated the truth of claims they had seen previously and claims they had not seen before. Of interest was (i) whether a single presentation during the exposure phase increases the acceptance of a given claim as true above baseline even when (ii) the claim contradicts participants’ pro-climate science attitudes. We found that this was the case. A single repetition increased perceived validity of all claim types, including claims that contradicted the participants’ pro-climate science attitudes. This held even for claims that participants themselves later classified as counter-attitudinal and even for the strongest climate science endorsers, those classified as “Alarmed” on the Six Americas Super Short Survey on climate change beliefs scale (SASSY) [40].

## Methods

### Power analysis and participant sample

The between-items effect size for the illusory truth effect is estimated as *d* = 0.49 95% CI [0.43, 0.57] [2,19]. Based on the smaller estimated effect in a meta-analysis [2], and assuming α = .05, power (1-β) = .95, and a two-tailed analysis, G*Power [40] indicates that 54 participants are required to detect a truth effect in a repeated measures design. Thus, to ensure sufficient power and high precision after allowing for exclusions, we posted 100 HITs in Experiment 1 and 200 HITs in Experiment 2.

For both experiments, we recruited US participants on Amazon’s online Mechanical Turk (MTurk; www.mturk.com/mturk) platform with a ≤ 95% HIT approval ratio and paid them $3.60 USD for completing the 30-minute experiment. When researchers follow best practice recommendations [41], MTurk participants are more attentive and reliable than participants in academic subject pools [42]. We used Qualtrics to randomly assign participants to counterbalances, and we downloaded data only after all available HITs were completed. The current studies were approved by the Human Research Ethics Committee at The Australian National University (protocol 2020/438). All participants provided informed consent before beginning the experiment by ticking a box to indicate that they had read through the participant information sheet and would like to participate in the experiment.

### Statistical methods

All statistical analyses were conducted using SPSS version 28.0 [43]. To investigate the effect of claim type on the size of illusory truth, in each experiment, we conducted a linear mixed effects model analysis with repetition (repeated vs. non-repeated) and claim type (scientist vs. skeptic) as fixed effects (using numerical effects coding), participant as a random effect, and perceived truth as the outcome variable. Further, in Experiment 2, we added participants’ general endorsement of climate change science as a covariate to the above model. The analyses were preregistered.

These linear mixed models are further supplemented by t-tests to examine differences between claim type (scientist/skeptic) and/or repetition (repeated/non-repeated), as a directional follow-up of main and interaction effects. We also present analyses of the main research question across various subsets of the full sample as categorized by climate change concern using SASSY [44], to look at the stronger endorsers of climate science. The linear mixed effects model is the overarching analysis for each experiment (and is consistent with pairwise comparisons).

All t-tests are within-subjects, reporting significance for the two-tailed test. We used paired-samples Cohen’s *d* using a corrected standard deviation of the difference to estimate effect size and corresponding confidence intervals across all t-tests, and report Hedges’ g_av_ which corrects for bias in *d*. We calculated effects sizes [45] in the linear mixed models using estimated means and SDs. For all tests, the threshold for significance was *p* = .05. For all bar graphs, error bars show 95% confidence intervals of each estimated cell mean.

To ensure that our data meet the assumptions for linear mixed models, we plotted residuals against predicted values to check for homogeneity of variance and created a histogram for residuals to check for normality of error term. We further examined assumptions for t-tests through inspecting a histogram of differences between pairs to check for normality of differences and extreme outliers. All assumptions were met for both experiments.

### Experiment 1

#### Participants

Of 100 HITS posted between the recruitment period from 28/09/2020 to 29/09/2020, we received 99 completed responses (65% identified as male, 35% identified as female, 0% other; aged 22–68; 95% American) with no missing data. As preregistered (https://aspredicted.org/blind.php?x=3jn4m8), we excluded 20 people who looked up the answers and 27 who failed the open-ended bot-check question. We retained 52 participants for analysis (59% identified as male, 41% identified as female, 0% identified as other/non-disclosed; aged 25–68; 95% American), exceeding the requirements of the power analysis.

#### Design, materials, and procedure

We used a fully within-subjects 2 (repetition: repeated, non-repeated) x 2 (claim type: scientist, skeptic) repeated-measures design.

For Experiment 1, we piloted an initial set of 42 climate-related claims that people may encounter in everyday life, asking people to categorize each claim as being consistent with views of a climate scientist, climate skeptic, or unknown. From these pilot data we selected 8 target claims that the majority of participants had classified as being consistent with views of a climate scientist and 8 target claims classified as consistent with a climate skeptic; 4 claims of each type were objectively true and 4 objectively false. We also piloted 32 weather claims with similar perceived validity (16 true and 16 false claims) to act as filler items. All claims and scales across both experiments are listed in S1 File.

To implement the standard ITE paradigm, we created two counterbalances of claims. Each counterbalance contained 4 scientist claims, 4 skeptic claims, and 8 filler weather claims, balanced in terms of perceived validity, objective validity, and topic. Inspecting either counterbalance separately did not affect the main findings.

Experiment 1 had two main sections. To capture the beliefs and attitudes of our sample, participants first answered an 11-item collection of 7-point Likert scales measuring belief and attitudes towards the science of climate change [46]. These scales comprised of climate-adapted versions of the Belief in Science Scale (e.g., “We can only rationally believe in what is scientifically provable about climate change”) and the Attitudes Towards the Scientific Method Scale (e.g., “The use of the scientific method to form opinions, make decisions, and better understand climate change is Worthless/Useful”).

Next, participants completed the classic illusory truth effect paradigm with three phases: encoding, delay, and test. Firstly, in the encoding phase, participants were told that they would see a series of trivia statements. Participants were exposed to one out of two counterbalanced subsets of claims (4 scientist, 4 skeptic, 8 weather). Each claim was shown for 8 seconds, individually in large black font on a white background. Participants then completed a delay task of 15 mins, where they read and answered questions about a passage on the topic of bread, and completed some visual rotation exercises. In the final test phase, participants rated the truth of all 32 claims (8 scientist, 8 skeptic, 16 weather claims), half of which they had seen before. Truth ratings were made on 6-point Likert scales (1 = Definitely True to 6 = Definitely False), which we recoded for analysis so that larger numerals reflect greater perceived truth.

All claims were presented in random order within the encoding and test phases, and we used two counterbalance groups (as described in the Materials section above) to ensure that each claim appeared equally often as repeated or non-repeated across participants. Thus, a claim may act as a repeated claim for one participant but appear as a non-repeated claim for another.

### Experiment 2

Experiment 2 is a replication of Experiment 1 with several minor changes. We i) doubled the number of participants to increase precision of measurement, ii) refined our set of target claims for objective validity, and iii) included an additional measurement of climate change attitudes which allowed greater discernment within climate science endorsers and skeptics (the Six Americas Super Short Survey [44]). In addition, iv) we asked participants to classify each claim as scientist or skeptic-aligning, thus providing a subjective measure of claim type that bears on whether participants perceived the claim as pro- or counter-attitudinal for themselves.

Results for both experiments are reported together in the results section below.

#### Participants

In Experiment 2 we doubled the number of HITs posted (N = 200) to increase precision of measurement. We received 197 completed responses (51% identified as male, 48% identified as female, 1% other; aged 23–75; 97% American) in the recruitment period from 03/06/2021 to 09/06/2021. To control for data quality, we excluded (preregistered at https://aspredicted.org/blind.php?x=vn5y53) 26 participants who looked up the answers and a further 51 participants who failed the bot-check question, leaving 120 responses for analysis (52% identified as male, 47% identified as female, 1% identified as other/non-disclosed; aged 23–75; 97% American).

#### Design, materials, and procedure

Experiment 2 had the same design as Experiment 1.

In Experiment 2, we further refined our target claims and piloted them again using the same category options as in Experiment 1. Drawing on the same selection criteria, we obtained a set of 8 target claims (4 climate scientist claims, 4 skeptic claims). Again, we created two matched counterbalances containing target climate-related claims, and weather claims from Experiment 1. Inspecting either counterbalance separately did not affect the main findings.

Experiment 2 followed the same procedure as Experiment 1 with two additions. After giving truth ratings of all claims in the truth testing phase (4 scientist, 4 skeptic, 16 weather), participants completed SASSY [44], a well-validated measure of climate change attitudes allowing categorical analysis of climate change position/beliefs, as an alternative measure of the between-subjects covariate of Belief. Then, participants self-classified each climate-related claim as aligning with views of a climate scientist, views of a climate skeptic, or if they were unsure.

## Results

### Sample characteristics

#### Most participants are climate science endorsers

Our key research question was whether a single repetition increases the perceived truth of a claim even when the claim runs counter to the recipient’s own climate change beliefs. Hence, we first examined the beliefs and attitudes of our participants across both Experiments 1 and 2. We calculated mean scores of the Belief in (climate change) Science (Cronbach’s alpha = .91 for Experiments 1 and 2) and Attitudes Towards (climate change) Science items (Cronbach’s alpha = .83 for Experiment 1, .81 for Experiment 2). We used participants’ mean score across both scales to represent the belief-in-climate-science variable. We found that 90% of participants in Experiment 1 and 92% in Experiment 2 endorsed anthropogenic climate change (having mean scale scores of above 4—the middle of the 7-point scale). This characterization of our samples was supported by using SASSY in Experiment 2: of our 120 participants (including 5 uncategorized), 36 participants were Alarmed (31%), 35 Concerned (29.2%), 27 Cautious (22.5%), 0 Disengaged (0%), 8 Dismissive (7%), and 14 Doubtful (11.7%). These samples allow us to examine how the repetition of climate skeptic-aligned claims affects the beliefs of climate science endorsers, which is central to the discussion of false-balance reporting [3–5]. However, our samples do not allow us to examine the response of climate skeptics to repetitions of climate science-aligned claims.

In our main analyses, we include only people who are categorized as climate science endorsers by the belief variable (Experiment 1; *N* = 47) or by both the belief variable and the SASSY classification (Experiment 2; *N* = 110). Accordingly, skeptic claims are always counter-attitudinal claims that contradict the climate change beliefs and attitudes of our samples. Including participants who do not endorse climate science in the linear mixed models does not change our main conclusions.

### Illusory truth effect for counter-attitudinal claims

#### Repetition increased truth ratings for both pro- and counter-attitudinal claims

Our main research question was whether climate science endorsers would perceive climate-skeptic claims as more likely to be true when they have seen them before. That is, can a single repetition of a counter-attitudinal claim increase its perceived truth? To answer this question, we tested whether claim type (climate scientist-aligned vs. climate skeptic-aligned) moderated the influence of repetition on judged truth by conducting a linear mixed effects model with the following factors: participant (random), claim type (fixed; scientist-aligned vs. skeptic-aligned), repetition (fixed; repeated vs. non-repeated), and repetition x claim type interaction (fixed). We report results for each fixed effect in order below, and also present estimates of fixed effects for Experiment 1 (Table 1) and Experiment 2 (Table 2).

**Table 1:** Estimates of fixed effects in Experiment 1 with endorsers only.

Variable	*B*	*SE*	df	*t*	*p*	95% CI
Intercept	2.88	.15	125.65	19.74	< .001	[2.59, 3.17]
Repetition [repeated]	.48	.15	701.03	3.19	.001	[.19, .78]
Claim Type [scientist-aligning]	.70	.15	701.09	4.62	< .001	[.40, 1.00]
Repetition [repeated] * Claim Type [scientist-aligning]	.25	.21	701.06	1.17	.24	[-.17, .67]

**Table 2:** Estimates of fixed effects in Experiment 2 with endorsers only.

Variable	*B*	*SE*	df	*t*	*p*	95% CI
Intercept	3.02	.12	446.78	26.08	< .001	[2.79, 3.24]
Repetition [repeated]	.63	.15	641.00	4.27	< .001	[.34, .92]
Claim Type [scientist-aligning]	.85	.15	641.00	5.74	< .001	[.56, 1.14]
Repetition [repeated] * Claim Type [scientist-aligning]	.11	.21	641.00	.520	.60	[-.30, .52]

Given that our samples were climate science endorsing, we would expect people to rate climate scientist-aligned claims as truer than skeptic-aligned claims. This is what we found. Across both experiments, there was a significant main effect of claim type, Experiment 1: *F*(1, 701) = 59.36, *p* < .001; Experiment 2: *F*(1, 641) = 74.58, *p* < .001. Comparison of raw *M* and *SD*s showed consistent findings across both experiments. In Experiment 1, scientist-aligned claims (*M* = 3.95, *SD* = .79) were rated as more true than skeptic-aligned claims (*M* = 3.13, *SD* = 1.01), *t*(46) = 5.94, p < .001, *d*_*z*_ = .89, 95% CI [.55, 1.25]. This pattern replicated in Experiment 2—climate scientist-aligned claims (*M* = 4.13, *SD* = .76) were again rated as more true than skeptic-aligned claims (*M* = 3.48, *SD* = 1.04), *t*(109) = 5.54, *p* < .001, *d*_*z*_ = .71, 95% CI [.44, .98].

**Fig 1:**
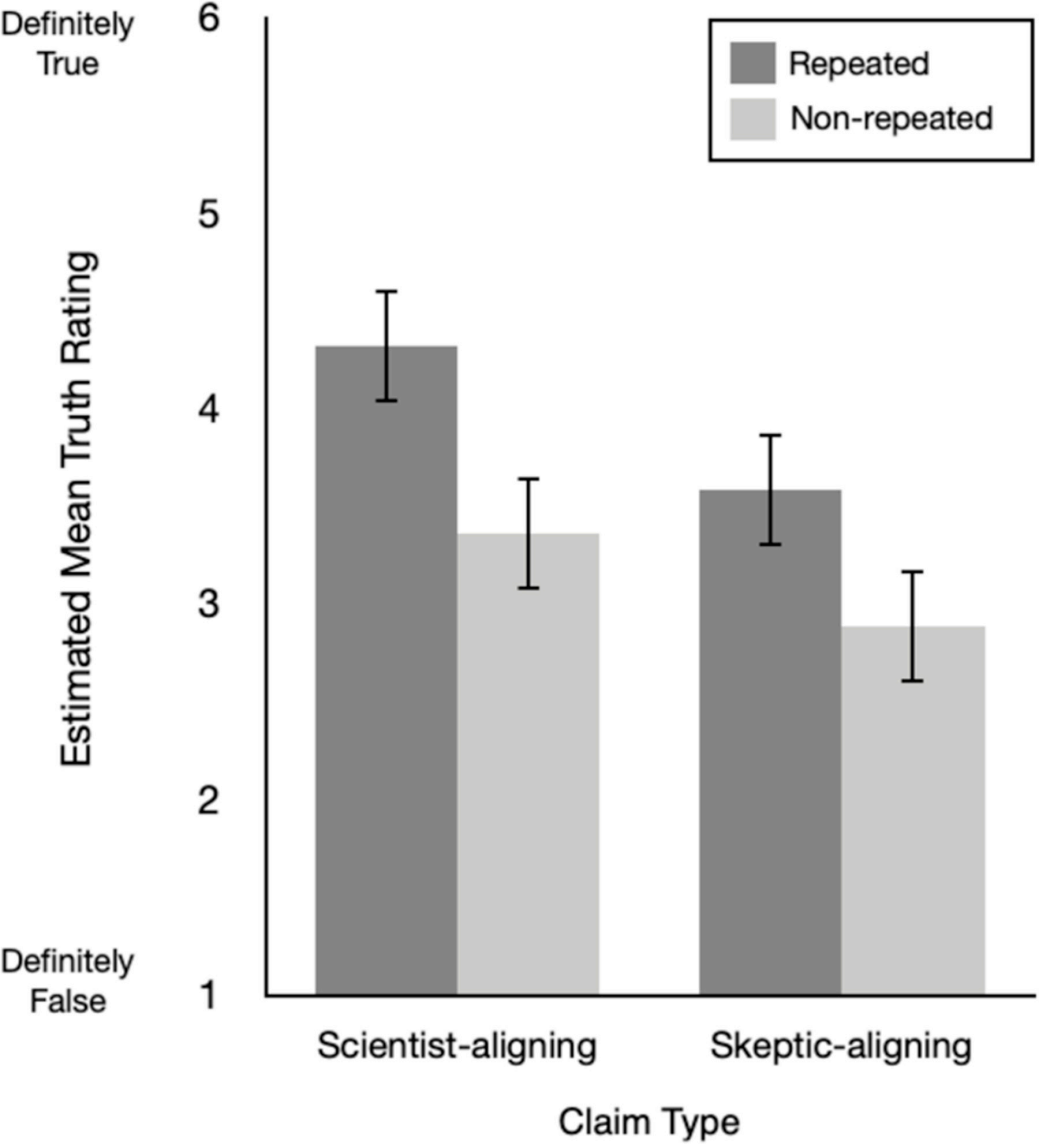
Estimated mean truth ratings across repetition (repeated, non-repeated) and claim type (science-aligning, skeptic-aligning) in Experiment 1. Note. Error bars show 95% CI.

**Fig 2:**
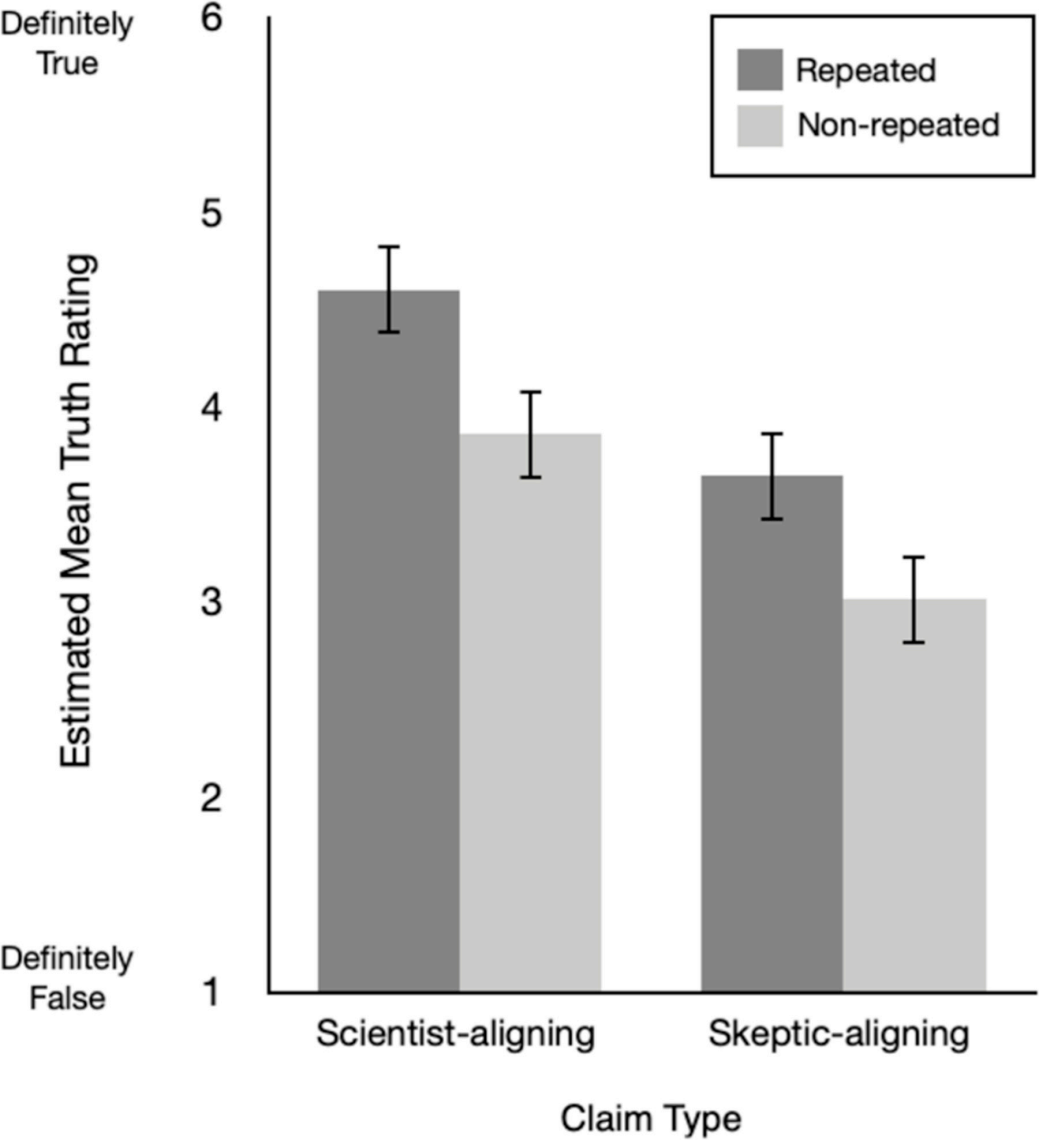
Estimated mean truth ratings across repetition (repeated, non-repeated) and claim type (science-aligning, skeptic-aligning) in Experiment 2. Note. Error bars show 95% CI.

Replicating the familiar ITE, people rated repeated claims as truer than non-repeated claims. Across both experiments, we found a significant main effect of repetition, Experiment 1: *F*(1, 701) = 32.34, *p* < .001; Experiment 2: *F*(1, 641) = 42.97, *p* < .001. Comparison of raw *M* and *SD*s showed consistent findings across both experiments. In Experiment 1, repeated claims (*M* = 4.08) were rated as more true than non-repeated claims (*M* = 3.30), *d*_*z*_ = .87, 95% CI [.48, 1.27]. In Experiment 2, repeated claims (*M* = 4.50) were again rated as more true than non-repeated claims (*M* = 3.59), *d*_*z*_ = 1.19, 95% CI [.90, 1.50].,

A significant interaction between repetition and claim type would indicate that the effect of repetition on truth assessments is moderated by whether the claim is counter-attitudinal or not. We did not find this effect in Experiment 1, *F*(1, 701) = 1.38, *p* = .24, or Experiment 2, *F*(1, 641) = .27, *p* = .60. Estimated mean truth ratings across both repetition and claim type are presented in Fig 1 (Experiment 1) and Fig 2 (Experiment 2). As an exploratory analysis of the size of ITE, we found a medium to large effect of repetition for scientist-aligning as well as skeptic-aligning claims in both experiments (Table 3). Taken together, these findings show that repetition increased perceived truth to a similar extent for each claim type, indicating that repetition is similarly influential for pro- and counter-attitudinal claims.

**Table 3:** Effect size calculations of the repetition effect across claim type in each Experiment.

	Repeated	Non-Repeated	*t*	*p*	*d*_*z*_	95% CI
Exp 1 Scientist-aligning	*M* = 4.32, *SD* = .97	*M* = 3.57, *SD* = .95	*t*(46) = 4.64	< .001	.76	[.41, 1.14]
Exp 1 Skeptic-aligning	*M* = 3.37, *SD* = 1.24	*M* = 2.88, *SD* = 1.02	*t*(46) = 3.19	< .001	.42	[.15, .70]
Exp 2 Scientist-aligning	*M* = 4.56, *SD* = 1.10	*M* = 3.70, *SD* = 1.08	*t*(109) = 5.76	< .001	.78	[.50, 1.06]
Exp 2 Skeptic-aligning	*M* = 3.78, *SD* = 1.39	*M* = 3.18, *SD* = 1.05	*t*(109) = 4.78	< .001	.49	[.28, .70]

In S2 File we also report pre-registered mixed models which include the factor of belief (measured through beliefs and attitudes scales in both experiments, and SASSY in Experiment 2) as a covariate. These analyses allowed us to inspect truth assessment across claim type and repetition while taking into account people’s variation across general endorsement of climate change science. These pre-registered mixed models report the same significant pattern of results with repetition, and no interaction between repetition x claim type, consistent with what we reported above.

#### Visual representation of the ITE across claim types

**Fig 3:**
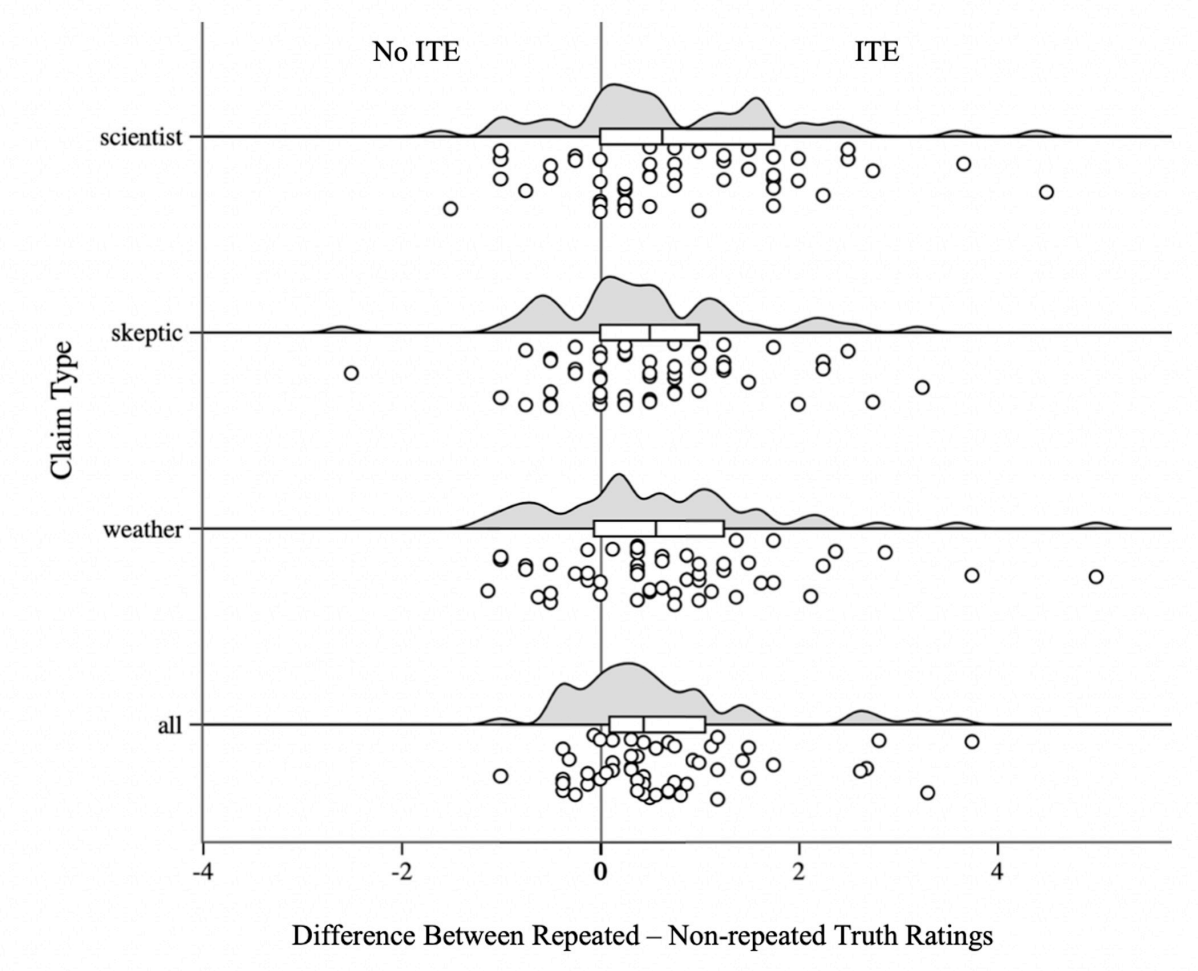
Raincloud plot of truth rating differences between repeated and non-repeated claims across claim types (all, scientist, skeptic, weather) in Experiment 1. Shown is the difference between the truth ratings when claims were repeated vs. not repeated, computed as (mean of repeated claims minus mean of non-repeated claims), for each participant. Values above zero reflect a repetition-induced truth effect, where on average repeated claims are rated as more true than non-repeated claims.

**Fig 4:**
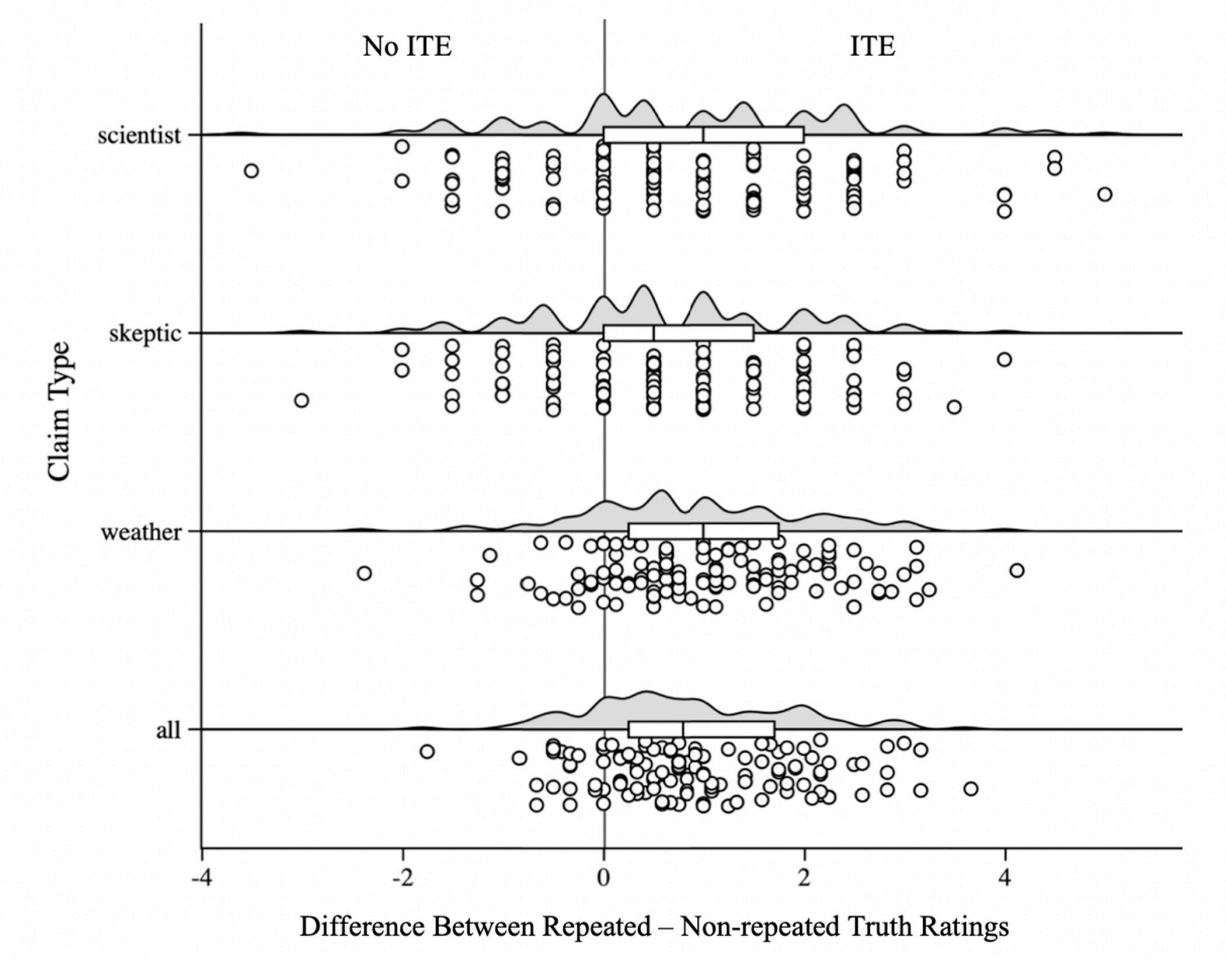
Raincloud plot of truth rating differences between repeated and non-repeated claims across claim types (all, scientist, skeptic, weather) in Experiment 2. Shown is the difference between the truth ratings when claims were repeated vs. not repeated, computed as (mean of repeated claims minus mean of non-repeated claims), for each participant. Values above zero reflect a repetition-induced truth effect, where on average repeated claims are rated as more true than non-repeated claims.

To visualize the magnitude of the repetition effect for each claim type, we calculated the difference in truth ratings between repeated and non-repeated claims for each claim type, presented in Fig 3 (Experiment 1) and Fig 4 (Experiment 2). As shown in these figures, the influence of prior exposure applies to all claims and its size is very similar across types of claims. For our climate science endorsing participants, a single exposure increased the perceived truth of climate scientist claims as well as the perceived truth of climate-skeptic claims. It also increased agreement with unrelated weather claims, resulting in a strong overall influence of repetition on all the claims shown.

Further, we present mean truth ratings for each skeptic claim acting as either a repeated or non-repeated claim to allow an inspection of possible item effects. In short, we find a consistent pattern in the effect of repetition for Experiment 1 (S1 Fig) and Experiment 2 (S2 Fig).

### Stronger tests of the ITE for counter-attitudinal claims

#### ITE held for subjectively counter-attitudinal claims

It is conceivable that some participants did not recognize our pilot-tested skeptic claims as skeptic-aligning and hence counter-attitudinal. To address this possibility, we asked participants in Experiment 2 to identify whether a claim aligns with climate science or climate skeptics. This allowed for an analysis where we treated the claims as scientist-aligned or skeptic-aligned based on each participant’s personal classification at the end of the study. We inspected only truth ratings for claims which participants self-classified as skeptic-aligning in a linear mixed model that parallels the previously used model (Table 4). This linear mixed-effects model included the following factors: participant (random), repetition (fixed; repeated vs. non-repeated), subjective claim type (fixed; scientist vs. skeptic), and repetition x claim type interaction (fixed).

**Table 4:** Estimates of fixed effects using subjective claim type in Experiment 2 with endorsers only.

Variable	*b*	*SE*	df	*t*	*p*	95% CI
Intercept	2.46	0.21	166.27	12.00	< .001	[2.05, 2.86]
Repetition [repeated]	0.68	0.26	218.85	2.61	0.01	[.17, 1.20]
Claim Type [scientist-aligning]	1.7	0.26	229.54	6.62	< .001	[1.20, 2.21]
Repetition [repeated] * Claim Type [scientist-aligning]	0.19	0.36	226.57	0.51	0.61	[-.53, .90]

Identical to results for the LMM using piloted claim type, we found a main effect for repetition, *F*(1, 216) = 18.78, *p* < .001, and a main effect for subjective claim type, *F*(1, 240) = 94.43, *p* < .001, but no interaction effect, *F*(1, 227) = .26, *p* = .61. The lack of an interaction in combination with a main effect of repetition suggests that people showed an ITE for both claim types. Indeed, even claims that participants self-classified as skeptic-aligning had higher estimated mean truth ratings when they were repeated (*M* = 3.14, *SE* = .20) than when they were not (*M* = 2.46, *SE* = .21), with a mean difference of .68, 95% CI [-.12, 1.49]. T-tests on the raw scores are not reported due to the difficult interpretation of low sample sizes. Estimated mean truth ratings across repetition and claim type are presented in S3 Fig.

#### ITE held for strongest endorsers of climate science

It is also conceivable that our participants endorsed climate science but were not particularly committed to these beliefs. To address this possibility, we examined whether the effect sizes for skeptic-aligning claims varied depending on how strongly participants endorsed climate science. To do so, we used empirically derived categories from the SASSY scale [44] in Experiment 2, which distinguishes groups of individuals based on their beliefs and behaviors regarding climate change. These groups range from the Alarmed group, who are the most concerned about climate change, to the Dismissive group, who reject that the climate problem is real. People with less extreme views belong to one of four other groups—the Concerned (who believe in climate change being a problem, but are less involved in action than the Alarmed), Cautious (who also believe that climate change is a problem, but are less certain and involved than the Concerned), Disengaged (who pay little attention to climate change), or Doubtful (who believe that climate change is naturally-caused and America is already doing enough to respond to climate change).

**Fig 5:**
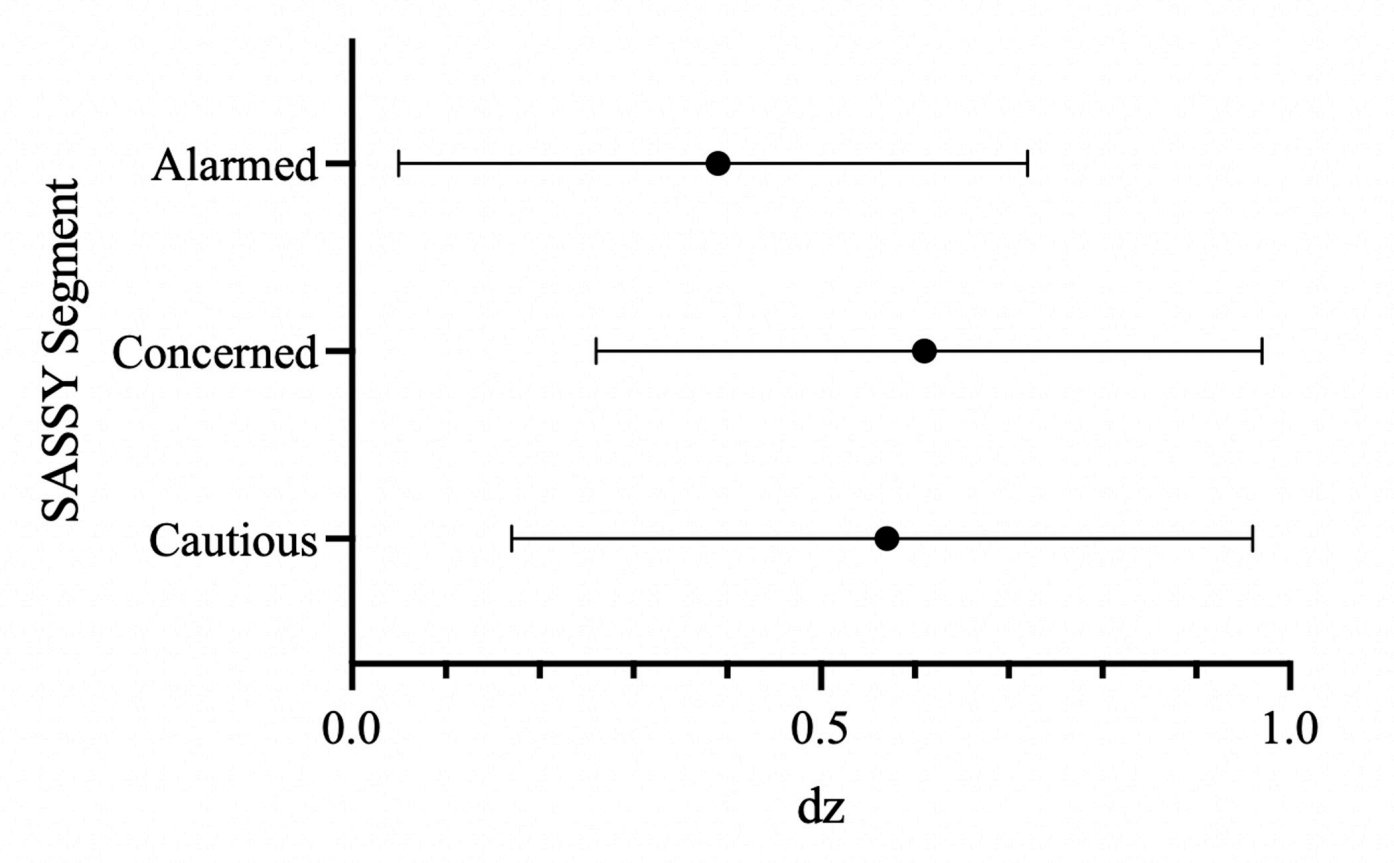
Forest plot of effect sizes (*d*_*z*_) of repetition for skeptic aligning claims across climate change-endorsing SASSY groups (Alarmed, Concerned, Cautious).

To investigate whether climate change endorsers are susceptible to the repetition of counter-attitudinal claims, we tested the effect of repeating skeptic claims for the Alarmed, Concerned, and Cautious groups, the three groups with the highest N in our sample. As shown in Fig 5, the effect sizes of repetition for skeptic-aligning claims were similar across the Alarmed, Concerned, and Cautious groups with overlapping 95% confidence intervals.

Specifically, those in the Cautious segment rated repeated skeptic-aligning claims (*M* = 3.94, *SD* = 1.15) as more true than non-repeated skeptic-aligning claims (*M* = 3.37, *SD* = .77), *t*(26) = 2.65, *p* = .013, with an effect size of *d*_*z*_ = .57, 95% CI [.17, .96]. Those in the Concerned segment similarly rated repeated skeptic-aligning claims (*M* = 3.64, *SD* = 1.39) as more true than non-repeated skeptic-aligning claims (*M* = 2.91, *SD* = .88), *t*(34) = 3.55, *p* = .001, with an effect size of *d*_*z*_ = .61, 95% CI [.26, .97]. Most importantly, even those in the Alarmed segment rated repeated skeptic-aligning claims (*M* = 3.50, *SD* = 1.50) as more true than non-repeated skeptic-aligning claims (*M* = 2.97, *SD* = 1.15), *t*(35) = 2.17, *p* = .037, with an effect size of *d*_*z*_ = .39, 95% CI [.05, .72]. Among the most fervent climate science supporters, the Alarmed segment, this translates into a 61% chance that a randomly sampled truth rating for repeated skeptic-aligning claims was higher than a randomly sampled truth rating for non-repeated skeptic claims.

As the strongest test of this question, we conducted a linear mixed effects model for only the Alarmed participants, with factors identical to the main reported model: participant (random), repetition (fixed; repeated vs. non-repeated), claim type (fixed; scientist vs. skeptic), and repetition x claim type interaction (fixed). Again, we found significant main effects of repetition, *F*(1, 249) = 16.91, *p* < .001 and claim type, *F*(1, 249) = 29.93, *p* < .001, but no interaction effect, *F*(1, 249) = .41, *p* = .52 (Table 5).

**Table 5:** Estimates of fixed effects in Experiment 2 for Alarmed group.

Variable	*b*	*SE*	df	*t*	*p*	95% CI
Intercept	2.89	0.21	155.87	13.79	< .001	[2.48, 3.30]
Repetition [repeated]	0.64	0.26	249.00	2.45	0.015	[.13, 1.15]
Claim Type [scientist-aligning]	0.89	0.26	249.00	3.42	< .001	[.38, 1.40]
Repetition [repeated] * Claim Type [scientist-aligning]	0.24	0.37	249.00	0.64	0.52	[-.49, .96]

In sum, our analyses establish that repetition increases perceived truth of counter-attitudinal claims for climate science endorsers, even when they themselves categorize the claims as counter-attitudinal, and even for the strongest endorsers of climate science, known as the “Alarmed” segment.

## General discussion

More than 90% of our participants endorsed climate science and were more inclined to believe climate scientist-aligned claims over skeptic or denial-aligned claims. Nonetheless, a single repetition was sufficient to increase the perceived truth of all claims–it made pro-attitudinal climate scientist-aligned claims seem more true and counter-attitudinal skeptic-aligned claims seem less false. In both experiments, repetition moved counter-attitudinal claims towards the midpoint of the scale, leading people to lean towards believing such claims. In combination, these findings highlight the benefits of repeating true information and the adverse consequences of repeating false information. It is therefore important to emphasize and repeat what is true and not to repeat what is false.

These findings are consistent with a growing body of research showing that people draw on both declarative information (the content of the claims) and experiential information (how easy it is to process the claims) in forming judgments of truth [6, 7, 13–15, 47, 48]. In the present studies, our climate science endorsing participants perceived claims aligned with climate scientists as more truthful than claims aligned with climate skepticism, a difference that reflects the content of the claims. However, both types of claims seemed truer when they were repeated than when they were not, reflecting the influence of repetition-based fluency. The influence of repetition held even for claims that participants self-classified as counter-attitudinal when we asked them to categorize each claim as climate scientist-aligning or climate skeptic-aligning (Experiment 2). When we used participants’ own classifications—thus ensuring that they knew a claim was skeptic-aligning—our climate science endorsing participants still considered repeated skeptic-aligning claims more truthful. This confirms that the influence of repetition on the acceptance of counter-attitudinal claims was not driven by misconceptions of the content of claims; instead, repeated exposure has a profound effect on people’s judgments even when they can personally identify a given claim as being at odds with their own position. Compatible with this conclusion, related work suggests that repeated exposure to fake news headlines featuring polarizing political figures increases acceptance of the fake news, even when the fake headline themes are likely at odds with the perceivers’ own political orientation [27]. Such pervasive effects are compatible with the observation that people have little insight into the influence of metacognitive experiences, in particular when those experiences are elicited by subtle incidental manipulations like repetition [12], color contrast [13], print fonts [49], or acoustic quality [7, 50]. This lack of insight limits people’s ability to protect against their impact, as indicated by the observation that individual differences in critical reasoning do not moderate the influence of claim repetition [51, 52].

Our findings have mixed implications for theories of motivated reasoning that assume that people with pre-existing beliefs treat pro-attitudinal information with a confirmation bias and counter-attitudinal information with a disconfirmation bias [29, 53–56]. Compatible with such theories, our participants perceived claims that were aligned with climate scientists as truer than claims aligned with climate skeptics. But challenging such theories, the motivated acceptance and rejection of claims was still susceptible to the effects of repetition. This observation is especially noteworthy because our experimental procedures rendered participants’ own pre-experimental attitudes highly salient—we asked them to rate their own attitudes towards climate science right before seeing any of the claims.

As Cotter and colleagues [57] noted in their discussion of boundary conditions of motivated reasoning, motivated biases are strongest under conditions of identity threat [58] and people are more receptive to counter-attitudinal information when self-affirmation procedures attenuate threats to the self [59, 60]. From this perspective, the exposure to a mix of counter-attitudinal and pro-attitudinal claims may not have been sufficiently threatening to elicit a strong disconfirmation bias. Future research may fruitfully explore whether a more confrontational presentation of counter-attitudinal claims renders repetition less influential. Blocking counter-attitudinal claims—rather than interspersing them with pro-attitudinal claims—may increase their threat effect, as may more extreme wordings or manipulations that highlight the identity relevance of one’s pre-existing attitudes. Further extensions of this research might also consider the combined effect of counter-attitudinal claims shared by low credibility sources as another possible condition that increases identity threat. While the illusory truth effect literature generally shows that repetition continues to increase perceptions of truth regardless of source information [21, 48] the combined effect of counter-attitudinal claims from low credibility sources, especially under conditions of political polarization, has not been tested.

### Implications for climate science communication

From the perspective of climate science communication, our results highlight the downside of repeating scientifically unsupported claims of climate skeptics: a single repetition is enough to nudge recipients towards acceptance of the repeated claim, even when their attitudes are aligned with climate science and they can correctly identify the claim as being counter-attitudinal. People may encounter claims in environments where veracity can be carefully inspected—hearing from a climate scientist in a lecture. However, social media and other online news outlets are a significant source of information, where people may encounter falsehoods decoupled from evidence and where claims may be shared repeatedly and rapidly, regardless of veracity. With the proliferation of climate science misinformation on social media [61] and the repetition of skeptic claims in the interest of allegedly “balanced” reporting [62], skeptic claims can affect not only recipients who may be predisposed to climate skepticism, but also recipients who are strong climate science supporters. While longitudinal analyses indicate that scientifically accurate media reporting has increased in recent years [63], there remains considerable variance across countries [64] and news outlets, with more conservative news sources providing less accurate coverage of the scientific consensus across a number of scientific topics [63].

Unfortunately, the large psychological literature on illusory truth effects shows that the power of repetition is robust over longer delays [65] and difficult to undermine—neither warning people that some of the claims they hear are false [19], nor individual differences in depth of thought or critical thinking fully eliminate the effect of repetition on assessments of truth [18, 51, 52], although such variables can (sometimes) attenuate the effect size. Fortunately, however, the power of repetition is not limited to skeptic-aligning information—it also extends to the repetition of scientifically correct information. Our participants rated claims that were aligned with climate scientists as truer when they were repeated than when they were not, even though most of our climate science-endorsing participants were already familiar with what they read. Moreover, the influence of repetition was independent of how strongly participants endorsed climate science. This implies that it is beneficial to repeat scientist-aligning information, even when recipients are already in agreement with it.

Finally, further research is warranted to better understand whether repeating counter-attitudinal information has similar effects on other samples, such as climate skeptics who were underrepresented in our samples, as well as for other topics where people hold divided beliefs and attitudes, such as immigration, education, and healthcare policies. It is also important to understand whether the effects hold over time, or with more repetitions. While ITE research demonstrates that one repetition of a trivia statement can lead to an increase in perceived truth a month after an initial exposure [65], with repeated exposures increasing perceived truth even up to 16 exposures later [66, 67], we do not know whether such persistent effects also emerge when the initial content is counter-attitudinal. For instance, under a motivated reasoning framework, people may seek out more evidence that is congruent with their beliefs [68] and regard such evidence as stronger than incongruent evidence [69], which over time may lead to more elaboration, higher accessibility, and more fluent processing of attitudinally congruent content.

In sum, the present results converge with insights from other content domains [8, 47] in supporting a straightforward communication recommendation: Do not repeat false information. Instead, repeat what is true and enhance its familiarity.

## Supporting information

S1 File Materials in Experiments 1 and 2: Scales and claims. (DOCX)

S2 File Linear mixed effects models in Experiments 1 and 2 with belief as covariate. (DOCX)

S3 File Experiment 1 dataset. (XLSX)

S4 File Experiment 2 dataset. (XLSX)

S1 Fig Mean truth ratings for repeated and non-repeated skeptic-aligning claims in Experiment 1. Note: large error bars are due to comparisons being between-subjects.
(TIF)

S2 Fig Mean truth ratings for repeated and non-repeated skeptic-aligning claims in Experiment 2. Note: large error bars are due to 95% CI comparisons being between-subjects and hence each bar has a lower *N*.
(TIF)

S3 Fig Estimated mean truth ratings across repetition (repeated, non-repeated) and subjective claim type (science-aligning, skeptic-aligning) in Experiment 2. (TIF)

S4 Fig Estimated mean truth ratings across repetition (repeated, non-repeated) and claim Type (science-aligning, skeptic-aligning) for alarmed group in Experiment 2. (TIF)
