# Supplementary material for: Repetition increases belief in climate-skeptical claims, even for climate science endorsers
Source: PLoS One. 2024 Aug 7;19(8):e0307294. doi: 10.1371/journal.pone.0307294 (PMC11305575; doi:10.1371/journal.pone.0307294)
Supplement: S1 File — (DOCX) [file pone.0307294.s001.docx]

**Materials**

**Experiment 1**

Belief in Climate Science survey statements: 1 (Strongly Disagree) to 7 (Strongly Agree)

1. We can only rationally believe in what is scientifically provable about climate change.
2. Science tells us everything there is to know about the reality of climate change.
3. All the climate change tasks human beings face are solvable by science.
4. The scientific method is the only reliable path to knowledge about climate change.
5. The only real kind of climate change knowledge we can have is scientific knowledge.
6. Science is the most efficient means of attaining truth about climate change.
7. Climate change scientists and science should be given more respect in modern society.

Attitudes Towards the Scientific Method:

1. To me, the use of the scientific method to form opinions, make decisions, and better understand climate change is: 1 (*Harmful*) to 7 (*Beneficial*)
2. To me, the use of the scientific method to form opinions, make decisions, and better understand climate change is: 1 (*Good*) to 7 (*Worthless*)
3. To me, the use of the scientific method to form opinions, make decisions, and better understand climate change is: 1 (*Pleasant*) to 7 (*Unpleasant*)
4. To me, the use of the scientific method to form opinions, make decisions, and better understand climate change is: 1 (*Worthless*) to 7 (*Useful*)

Objectively True Scientist-Aligning Claims

1. Climate change models successfully reproduce global land, air, and ocean temperatures since 1900.
2. Antarctica is gaining sea ice but losing land ice at an accelerating rate.
3. The hottest years on record globally are 2005 and 2010.
4. Sea levels around the world have risen nearly 20cm in the last 120 years.

Objectively False Scientist-Aligning Claims

1. The Earth is expected to warm 18°F (10°C) by the end of the century.
2. The golden toad was the first species to have gone extinct due to climate change.
3. Nuclear power generation contributes to global warming.
4. In terms of contribution to global warming, pork is the most environmentally dangerous food.

Objectively True Skeptic-Aligning Claims

1. Previous periods have been warmer than the present despite CO2 levels being lower than they are now.
2. Global warming will not increase the risk of skin cancer in human beings.
3. Urban heat has not significantly influenced temperature records over the 20th century.
4. Cold weather has nothing to do with the long-term trend of increasing global temperatures.

Objectively False Skeptic-Aligning Claims

1. Surface air temperatures were the same from the first decade of the twenty-first century (2000-2009) to the last decade of the twentieth century (1990-1999).
2. Emails seized from prominent climate scientists suggest conspiracy and data manipulation.
3. Capitalism decreases human involvement in climate change.
4. Global sea level has been rising at the same rate for the past 2000 years.

Objectively True Weather Related Claims

1. Hurricanes and tropical storms have been officially named since the 1950s.
2. The wind only makes a sound when it blows against an object.
3. About 1800 thunderstorms are occurring on earth at any given time.
4. The fastest speed a falling raindrop can hit you is 18mph.
5. The lowest temperature recorded on earth is -128.5°F (-89.2°C).
6. Cumulonimbus clouds hold up to half a million tons of water.
7. The largest hailstone recorded weighed 2.25lbs.
8. Port Martin, Antarctica has an average wind speed of 40 mph, and experiences gale force winds for over a hundred days a year.

Objectively False Weather Related Claims

1. Pinecone scales open when a storm is on the way.
2. Meteorologists can track weather but their predictions are mainly guesswork.
3. The first daily weather forecast ever printed appeared in the London Times in 1901.
4. Warm air sinks and then rises as it cools down causing wind.
5. Not all thunderstorms contain lightning.
6. The name of the instrument used to measure windspeed is a tellurometer.
7. Niagara falls has never frozen completely solid.
8. The stock market is unlikely to be affected by weather predictions.

**Experiment 2**

Belief and Attitudes Scales are identical to those in Experiment 1.

Six Americas Super Short Survey:

1. How important is the issue of global warming to you personally?

- Extremely important
- Very important
- Somewhat important
- Not too important
- Not at all important

1. How worried are you about global warming?

- Very worried
- Somewhat worried
- Not very worried
- Not at all worried

1. How much do you think global warming will harm you personally?

- A great deal
- A moderate amount
- Only a little
- Not at all
- Don’t know

1. How much do you think global warming will harm future generations of people?

- A great deal
- A moderate amount
- Only a little
- Not at all
- Don’t know

Objectively True Scientist-Aligning Claims

1. Climate change models successfully reproduce global land, air, and ocean temperatures since 1900.
2. The hottest years on record globally are 2016 and 2020.

Objectively False Scientist-Aligning Claims

1. The Earth is expected to warm 18°F (10°C) by the end of the century.
2. An individual's annual carbon footprint for motor vehicles is two times greater compared to their annual carbon footprint for airplane flights.

Objectively True Skeptic-Aligning Claims

1. Climate scientists fail to make reliable predictions of weather.
2. Different climate models often predict vastly different global warming outcomes.

Objectively False Skeptic-Aligning Claims

1. Global sea level has been rising at the same rate for the past 2000 years.
2. Emails seized from prominent climate scientists suggest conspiracy and data manipulation.
