# Supplementary material for: Repetition increases belief in climate-skeptical claims, even for climate science endorsers
Source: PLoS One. 2024 Aug 7;19(8):e0307294. doi: 10.1371/journal.pone.0307294 (PMC11305575; doi:10.1371/journal.pone.0307294)
Supplement: S2 File — (DOCX) [file pone.0307294.s002.docx]

As pre-registered, we report the full linear mixed effects models with the following factors: participant (random), repetition (fixed; repeated, non-repeated), claim type (fixed; scientist-aligning, skeptic-aligning), repetition x claim type interaction (fixed), and belief in climate science (Beliefs) as a covariate, measured either through the BISS and ATSM scales with a mean score named AllScience (Exp 1: S1 Table; Exp 2: S2 Table), or through SASSY (Exp 2: S3 Table). We have added Beliefs as a covariate in our models in order to control for people’s individual differences in climate change beliefs while we test for an influence of repetition and claim type on truth ratings. Repetition and claim type again used effects coding, and we standardized the Beliefs variable. We found converging results in all LMEMs across the two experiments when we put in Beliefs as a fixed effect instead of a covariate: when our manipulation of claim type is crossed with climate science belief, beliefs explain much of the variance in the model.

**S1 Table. Fixed-Effects Table from LMEM in Experiment 1 with Belief Measured Through AllScience.**

| Variable | *b* | *SE* | *t* | *p* | 95% CI |
| --- | --- | --- | --- | --- | --- |
| Intercept | 3.54 | .11 | 32.64 | <.001 | [3.32, 3.75] |
| Repetition [repeated] | .33 | .05 | 6.62 | <.001 | [.24, .44] |
| Claim Type [science aligning] | .34 | .05 | 6.83 | <.001 | [.25, .44] |
| Belief | .02 | .11 | .15 | .88 | [-.20, .23] |
| Repetition [repeated] * Claim Type [science aligning] | .07 | .05 | 1.38 | .17 | [-.03, .17] |
| Claim Type [science aligning] * Belief | .29 | .05 | 5.75 | <.001 | [.19, .39] |
| Repetition [repeated] * Belief | -.05 | .05 | -.97 | .33 | [-.15, .05] |

Note: the p value of repetition = .03 in the main fixed effects model.

**S2 Table. Fixed-Effects Table from LMEM in Experiment 2 with Belief Measured Through AllScience.**

| Variable | *b* | *SE* | *t* | *p* | 95% CI |
| --- | --- | --- | --- | --- | --- |
| Intercept | 3.80 | .06 | 63.25 | <.001 | [3.68, 3.92] |
| Repetition [repeated] | .38 | .05 | 8.28 | <.001 | [.29, .48] |
| Claim Type [science aligning] | .30 | .05 | 6.53 | <.001 | [.21, .39] |
| Belief | -.09 | .06 | -1.41 | .16 | [-.20, .03] |
| Repetition [repeated] * Claim Type [science aligning] | .06 | .05 | 1.37 | .17 | [-.03, .16] |
| Claim Type [science aligning] * Belief | .20 | .05 | 4.31 | <.001 | [.11, .29] |
| Repetition [repeated] * Belief | -.04 | .05 | -.94 | .35 | [-.14, .05] |

**S3 Table. Fixed-Effects Table from LMEM in Experiment 2 with Belief Measured Through SASSY.**

| Variable | *b* | *SE* | *t* | *p* | 95% CI |
| --- | --- | --- | --- | --- | --- |
| Intercept | 3.72 | .20 | 18.44 | <.001 | [3.32, 4.12] |
| Repetition [repeated] | .81 | .15 | 5.37 | <.001 | [.51, 1.10] |
| Claim Type [science-aligning] | -.13 | .15 | -.83 | .41 | [-.42, .17] |
| Belief | .16 | .36 | .44 | .66 | [-.56, .87] |
| Belief [SASSY = Alarmed] | -.01 | .23 | -.02 | .99 | [-.46, .45] |
| Belief [SASSY = Cautious] | .13 | .24 | .54 | .59 | [-.37, .55] |
| Belief [SASSY = Concerned] | .09 | .23 | .40 | .69 | [-.37, .55] |
| Belief [SASSY = Dismissive] | .35 | .31 | 1.12 | .27 | [-.27, .96] |
| Repetition [repeated] * Claim Type [science aligning] | .06 | .05 | 1.40 | .16 | [-.03, .15] |
| Repetition [repeated] * Belief | -.68 | .27 | -2.53 | .01 | [-1.21, -.15] |
| Repetition [repeated] * Belief [SASSY = Alarmed] | -.43 | .17 | -2.49 | .01 | [-.77, -.09] |
| Repetition [repeated] * Belief [SASSY = Cautious] | -.47 | .18 | -2.62 | .009 | [-.82, -.14] |
| Repetition [repeated] * Belief [SASSY = Concerned] | -.48 | .17 | -2.79 | .005 | [-.82, -.14] |
| Repetition [repeated] * Belief [SASSY = Dismissive] | -.40 | .23 | -1.73 | .08 | [-.86, .054] |
| Claim Type [science aligning] * Belief | .20 | .27 | .74 | .46 | [-.33, .73] |
| Claim Type [science aligning] * Belief [SASSY = Alarmed] | .63 | .17 | 3.66 | <.001 | [.29, .97] |
| Claim Type [science aligning] * Belief [SASSY = Cautious] | .36 | .18 | 1.98 | .048 | [.003, .71] |
| Claim Type [science aligning] * Belief [SASSY = Concerned] | .66 | .17 | 3.83 | <.001 | [.32, 1.001] |
| Claim Type [science aligning] * Belief [SASSY = Dismissive] | -.50 | .23 | -2.16 | .031 | [-.95, -.045] |

*Note*. The outcome variable is perceived truth rating.

These full LMEMs include all one-way and two-way interactions between repetition, claim type, and belief in climate science (the three-way interaction did not reach significance, thus it was removed from the final model). Results showed a consistently significant main effect of repetition in Experiment 1 with AllScience, *F*(1, 774) = 4.72, *p* = .03, Experiment 2 with AllScience, *F*(1, 835) = 5.81, *p* = .016, and Experiment 2 with SASSY, *F*(1, 827) = 44.38, *p* < .001. Estimated means (S4 Table) show a standard repetition-induced truth effect where repeated claims were perceived to be more true than non-repeated claims.

The main effect of claim type was significant in Experiment 1, *F*(1, 774) = 19.32, *p* < .001, and Experiment 2, *F*(1, 835) = 9.41, *p* = .002, where beliefs was measured through AllScience, but non-significant in the model where Beliefs was measured through SASSY, *F*(1, 827) = 2.79, *p* = .095. Estimated means (S4) show a tendency to rate scientist-aligning claims as more true. This main effect was qualified by a significant interaction effect between claim type x belief across Experiment 1 with AllScience, *F*(1, 774) = 33.09, *p* < .001, Experiment 2 with AllScience, *F*(1, 835) = 18.53, *p* < .001, and Experiment 2 with SASSY, *F*(5, 827) = 10.13, *p* < .001. Estimated means (S5 Table) reveal that across all models, the difference across claim type increased for stronger endorsers of climate science.

These models show that repetition, claim type, and beliefs consistently explain final assessments of truth. Importantly, the lack of a repetition x claim type interaction is consistent with main conclusions in the manuscript where the repetition-induced truth effect is not moderated by claim type.

**S4 Table. Estimated Mean Truth Ratings Across Repetition (Repeated, Non-Repeated) and Claim Type (Science-aligning, Skeptic-aligning) from LMEMs with Beliefs as Covariate Across Experiments 1 and 2.**

|  | Repeated claims | Non-repeated claims | Scientist-aligning claims | Skeptic-aligning claims |
| --- | --- | --- | --- | --- |
| Experiment 1 with AllScience | *M* = 3.87,  *SE* = .12 | *M* = 3.20,  *SE* = .12 | *M* = 3.88,  *SE* = .12 | *M* = 3.19,  *SE* = .12 |
| Experiment 2 with AllScience | *M* = 4.18,  *SE* = .076 | *M* = 3.42,  *SE* = .076 | *M* = 4.10,  *SE* = .076 | *M* = 3.50,  *SE* = .076 |
| Experiment 2 with SASSY | *M* = 4.23,  *SE* = .099 | *M* = 3.44,  *SE* = .099 | *M* = 3.94,  *SE* = .099 | *M* = 3.74,  *SE* = .099 |

**S5 Table. Fixed-Effects Table from LMEM In Experiment 2 with Subjective Claim Type.**

|  |  | Scientist-aligning claims | Skeptic-aligning claims | Mean difference |
| --- | --- | --- | --- | --- |
| Experiment 1 with AllScience | Low belief | *M* = 3.57,  *SE* = .17 | *M* = 3.47,  *SE* = .17 | .10 |
|  | High belief | *M* = 4.19,  *SE* = .17 | *M* = 2.92,  *SE* = .17 | 1.27 |
| Experiment 2 with AllScience | Low belief | *M* = 3.99,  *SE* = .11 | *M* = 3.78,  *SE* = .11 | .21 |
|  | High belief | *M* = 4.22,  *SE* = .11 | *M* = 3.21,  *SE* = .11 | 1.01 |
| Experiment 2 with SASSY | Dismissive group | *M* = 3.44,  *SE* = .30 | *M* = 4.69,  *SE* = .30 | -.25 |
|  | Alarmed group | *M* = 4.22,  *SE* = .14 | *M* = 3.21,  *SE* = .14 | 1.01 |
