# Supplementary figures and images for: Repetition increases belief in climate-skeptical claims, even for climate science endorsers
Source: PLoS One. 2024 Aug 7;19(8):e0307294. doi: 10.1371/journal.pone.0307294 (PMC11305575; doi:10.1371/journal.pone.0307294)

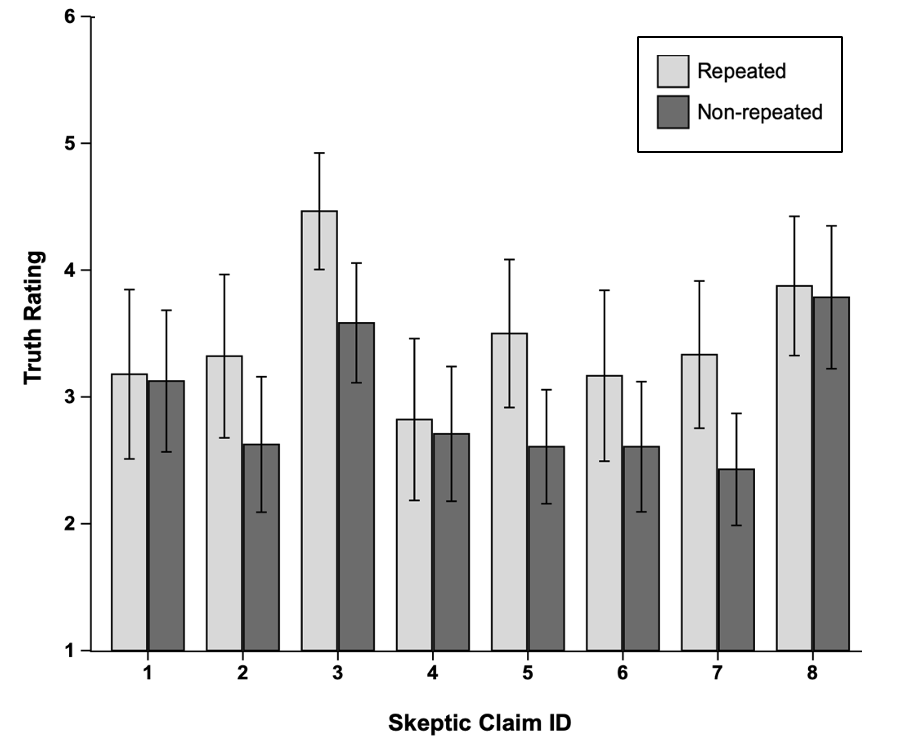

Supplement: S1 Fig — Note: large error bars are due to comparisons being between-subjects. (TIF) [file pone.0307294.s005.tif]

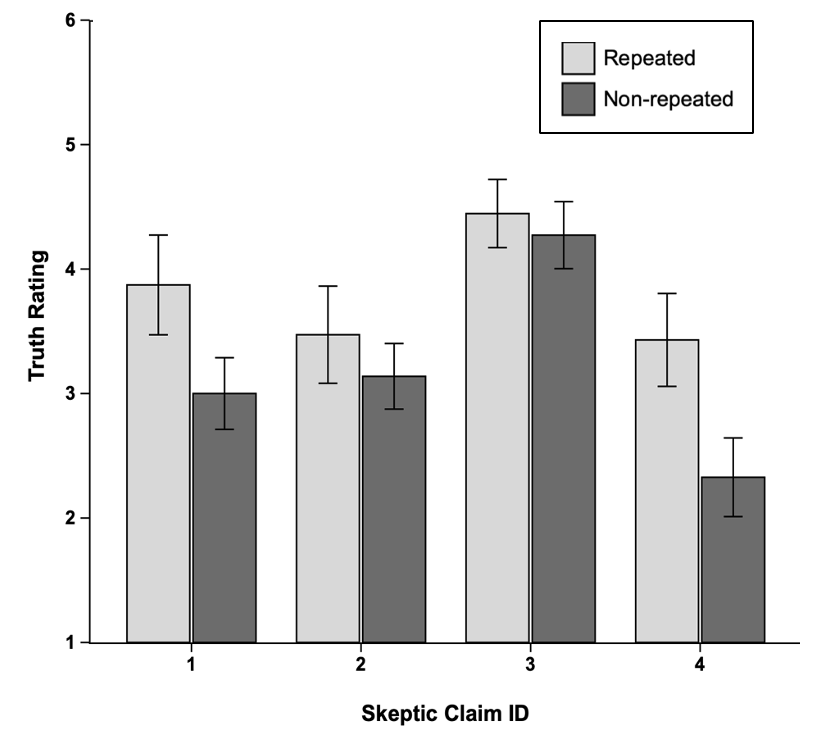

Supplement: S2 Fig — Note: large error bars are due to 95% CI comparisons being between-subjects and hence each bar has a lower N. (TIF) [file pone.0307294.s006.tif]

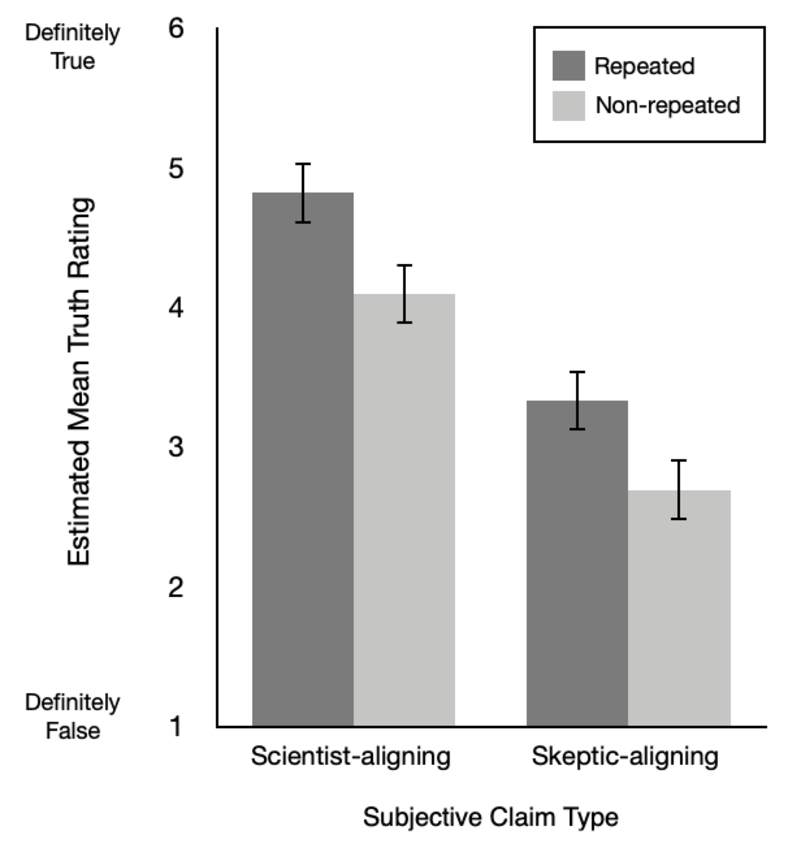

Supplement: S3 Fig — (TIF) [file pone.0307294.s007.tif]

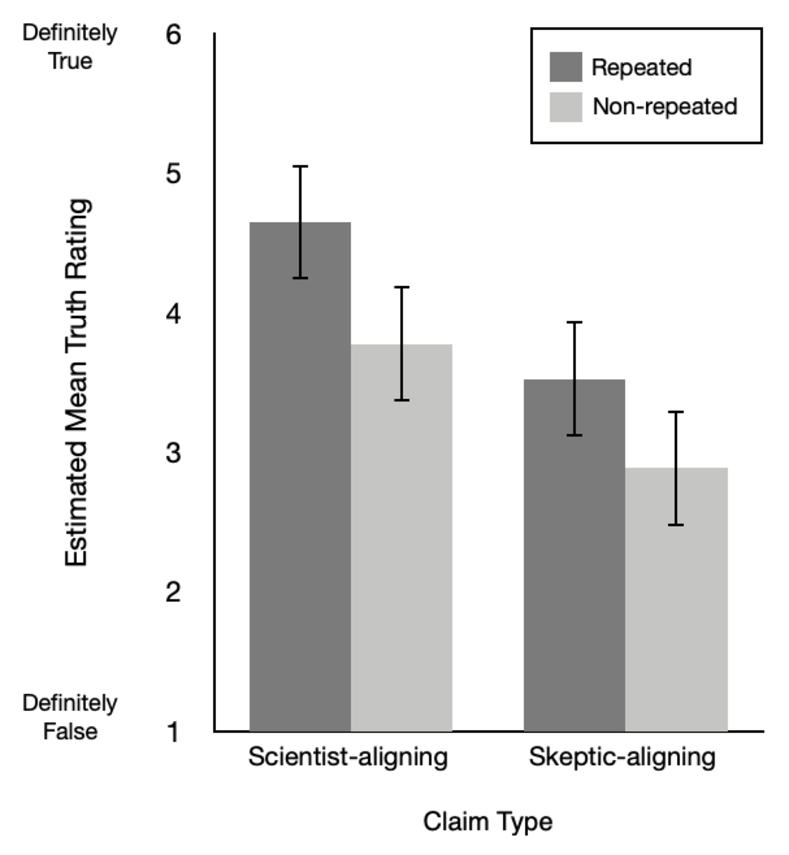

Supplement: S4 Fig — (TIF) [file pone.0307294.s008.tif]
